# Unveiling the m6A Methylation Regulator Links between Prostate Cancer and Periodontitis by Transcriptomic Analysis

**DOI:** 10.1155/2022/4030046

**Published:** 2022-09-12

**Authors:** Dexin Ding, Guobin Liu, Jianing Gao, Muyang Cao

**Affiliations:** Department of Urology Surgery, Harbin Medical University Cancer Hospital, Harbin 150081, China

## Abstract

**Objective:**

To identify the N6-methyladenosine (m6A) methylation regulator genes linking prostate adenocarcinoma (PRAD) and periodontitis (PD).

**Materials and Methods:**

PD and TCGA-PRAD GEO datasets were downloaded and analyzed through differential expression analysis to determine the differentially expressed genes (DEGs) deregulated in both conditions. Twenty-three m6A RNA methylation-related genes were downloaded in total. The m6A-related genes that overlapped between PRAD and PD were identified as crosstalk genes. Survival analysis was performed on these genes to determine their prognostic values in the overall survival outcomes of prostate cancer. The KEGG pathways were the most significantly enriched by m6A-related crosstalk genes. We also performed lasso regression analysis and univariate survival analysis to identify the most important m6A-related crosstalk genes, and a protein-protein interaction (PPI) network was built from these genes.

**Results:**

Twenty-three m6A methylation-related regulator genes were differentially expressed and deregulated in PRAD and PD. Among these, seven (i.e., *ALKBH5*, *FMR1*, *IGFBP3*, *RBM15B*, *YTHDF1*, *YTHDF2*, *and ZC3H13*) were identified as m6A-related cross-talk genes. Survival analysis showed that only the *FMR1* gene was a prognostic indicator for PRAD. All other genes had no significant influence on the overall survival of patients with PRAD. Lasso regression analysis and univariate survival analysis identified four m6A-related cross-talk genes (i.e., *ALKBH5*, *IGFBP3*, *RBM15B*, and *FMR1*) that influenced risk levels. A PPI network was constructed from these genes, and 183 genes from this network were significantly enriched in pathogenic *Escherichia coli* infection, p53 signaling pathway, nucleocytoplasmic transport, and ubiquitin-mediated proteolysis.

**Conclusion:**

Seven m6A methylation-related genes (*ALKBH5*, *FMR1*, *IGFBP3*, *RBM15B*, *YTHDF1*, *YTHDF2*, and *ZC3H13*) were identified as cross-talk genes between prostate cancer and PD.

## 1. Introduction

Prostate cancer (PC) is the most common kind of cancer in men worldwide. Epidemiological studies have identified several factors, such as age, ethnicity, family history, lifestyle, diet, environmental exposures, and occupational factors, that increase the risk for PC [1]. Periodontitis (PD) is a novel risk factor that is increasingly gaining attention among dental and urological researchers. A 2021 meta-analysis summarized nine cohort studies and concluded that periodontal disease raised the incidence of PC by 1.40-fold [2]. However, the underlying mechanisms linking periodontal disease and PC have not been extensively explored.

Previous researchers hypothesized that PC and PD are linked by periodontal pathogens and proinflammatory mediators, such as cytokines and chemokines, that are produced as a host response to PD [3]. A recent study identified *Fusobacterium nucleatum*, a periodontal pathogen, in prostate gland samples that showed signs of prostatitis and PC. However, more common periodontal pathogens, such as *Porphyromonas gingivalis*, *Prevotella intermedia*, *Treponema denticola*, *Tannerella forsythia*, and *Campylobacter rectus*, were not found in the same samples [4]. The identification of a periodontal-specific microorganism, as well as other inflammatory mediators, in prostate tissue samples indicates that these compounds can migrate from one site to another through the systemic circulation. The presence of foreign pathogens and inflammatory mediators in the prostate gland resulted in chronic prostatic inflammation [3], which may have contributed to a tumor microenvironment (TME) and PC initiation and progression [5].

In recent years, N6-methyladenosine (m6A) has gained increasing attention because of its role in dynamic regulation and reversible posttranscriptional regulation [6]. The RNA methylation of m6A occurs through methyltransferases (writers) and demethylases (erasers and readers), which are corresponding enzymes [7]. Emerging evidence has shown that alterations in m6A mRNA methylation may result in carcinogenesis and metastasis [8], as well as inflammatory diseases [9]. While there is a clear relationship between m6A RNA methylation and cancer and inflammation, an important question remains. Does m6A RNA methylation modification play a role in the pathogenesis of two related diseases? We hypothesize that regulators of m6A RNA methylation may link PC and PD on a genetic level.

We conducted an integrated analysis of both diseases by studying the genetic data available on the GEO [10] and TCGA databases [11]. Identifying the genetic links between two related diseases is possible because of the advent of computational biology and the analytical approach. Several bioinformatics studies have attempted to identify the cross-talk genes that are deregulated in PC and PD [12–17]. The present research similarly aims to identify the m6A RNA methylation regulators linking PC and PD.

## 2. Material and Methods

### 2.1. Study Flowchart

**Figure 1:**
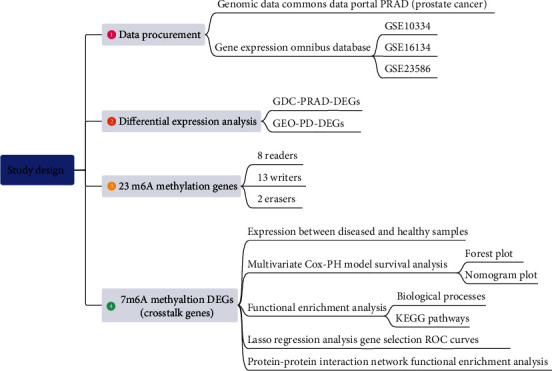
The schematic diagram of the current research.

Figure 1 illustrates the study design of the current research. Briefly, prostate cancer data and periodontitis data were obtained from the Genomic Data Commons (GDC) data portal and the Gene Expression Omnibus (GEO) database, respectively. The differential expression analysis was performed to identify the differentially expressed genes (DEGs) which were deregulated in prostate cancer and periodontitis, respectively. Afterward, 23 m6A methylation regulator genes were obtained and integrated with differentially expressed genes of both diseases, and thereby 7 m6A methylation DEGs were obtained and regarded as cross-talk genes. The subsequent analysis was based on these 7 cross-talk genes from some aspects including survival analysis, functional enrichment analysis, gene selection analysis, and protein-protein interaction network analysis.

### 2.2. Downloading Data

The RNA-seq dataset of prostate adenocarcinoma (PRAD) were downloaded from the Genomic Data Commons (GDC) data portal (https://portal.gdc.cancer.gov/) [18]. At the same time, we downloaded the clinical information of the relevant samples for PRAD. The last number of the sample ID between 01 and 10 is the disease group, and greater than 10 is the control group.

Gene expression profiling data for periodontitis in humans was downloaded from the GEO database (http://www.ncbi.nlm.nih.gov/) [19]. Samples from gingival tissue were selected, and both chronic and aggressive periodontitis were regarded as the disease group, and healthy was regarded as the control group. In order to reduce the data error caused by the platform, all the analysis data of periodontitis were downloaded from the chip data. Finally, three datasets of periodontitis were obtained: GSE10334 [20], GSE16134 [21, 22], and GSE23586 [23]. Finally, the statistics of the filtered datasets are shown in Table 1. The 23 m6A RNA methylation-related genes were acquired from the previous related research [24–27], including 8 readers, 13 writers, and 2 erasers (Table 2).

### 2.3. Data Preprocessing

For the PRAD obtained from the TCGA database, the ensemble ID was converted to gene symbol. The annotation file was downloaded from GENCODE (https://www.gencodegenes.org/human/) [28], then extracted the mapping information of Gene Symbol and ENSG_ID, and finally mapped ENSG_ID to Gene Symbol. When there were multiple matches, the median was taken, and the transformed expression profile was finally obtained. Since the expression value type is HTSeq Counts, the two datasets were merged together based on their overlap genes, and therefore, 496 cancer samples and 52 healthy samples were acquired finally.

For the microarray dataset obtained from the GEO database, the probes were converted into corresponding gene names based on the platform information of GPL570. When multiple probes corresponded to the same gene, the mean of the expression values of these probes in a certain sample were selected as the expression value of the gene in this sample. Then, according to the overlapped genes appearing in the three datasets of PD, all the samples under the three datasets were merged together. After merging, in order to reduce the difference caused by the merging of samples from different batches, ComBat method in the sva package (version 3.15) [29] of R was used to perform batch correction on the merged data. Finally, 427 PD disease samples and 136 healthy samples were acquired. For PRAD and PD datasets, the genes from the dataset whose expression value is 0 in more than half of the samples were deleted.

### 2.4. Differentially Expressed Gene Analysis

For the PD dataset, the “limma” package (version 3.15) of R project [30] was used to perform the differentially expressed gene analysis, and genes with *p* value<0.05, |log2(FC)| ≥1 were differentially expressed genes. For the PRAD, the “ limma” package of R project was used for analysis, and genes with *p* value<0.01, |log2(FC)| ≥1 were selected as differentially expressed genes.

### 2.5. The m6A-Related Cross-Talk Gene between in PRAD and PD

To identify the m6A-related cross-talk genes between PARD and PD, the m6A-related genes, DEGs of PRAD and DEGs of PD, were merged together. The common genes among m6A-related genes, DEGs of PRAD and DEGs of PD, were the potential m6A-related cross-talk genes between PRAD and PD. Finally, 7 genes (ALKBH5, FMR1, IGFBP3, RBM15B, YTHDF1, YTHDF2, and ZC3H13) were acquired.

### 2.6. Tumor Mutation Burden (TMB) Analysis of m6A-Related Cross-Talk Genes in PRAD

Tumor mutational burden (TMB) refers to the total number of somatic mutations per Mb base in the exon coding region of the genome in a tumor sample. The more mutated genes in tumor tissue, the more likely it is to produce more abnormal proteins, and tumors may also have a greater impact on the whole body. At the same time, the more easily these abnormal proteins are recognized by the immune system, thereby activating the body's anticancer immune response, so the efficacy of tumor immunotherapy is better. By calculating the Simple Nucleotide Variation dataset downloaded by TCGA, the TMB score for each sample in PRAD was obtained. According to the TMB score, all samples were divided into a high score group and a low score group according to the median of TMB score. After grouping the samples, survival analysis was performed on the samples based on clinical information to see the 3-year survival outcome. Meanwhile, the expression values of 7 different m6A key genes in PRAD samples were extracted, and the correlation analysis of these genes was conducted based on Pearson's coefficient combined with TMB scores.

### 2.7. Survival Analysis of m6A Cross-Talk Gene Was Performed

Since there was no survival data for periodontitis, the survival risk of 7 m6A cross-talk genes in PRAD was analyzed. First, we extracted the expression values of 7 m6A cross-talk genes in PRAD tumor samples. Then, each gene was regarded as a variable, and a Cox proportional risk regression model (COX-PH) [31] was established for each variable using the “survival” package (version 3.4-0) [32] of R project to perform the univariate analysis. Based on a univariate COX-PH model, the risk score for each gene for all tumor samples was obtained, and then the samples into the high-risk and low-risk groups were divided based on the median risk score. “survival” package (version 3.4-0) of R project was used for survival analysis of the two risk groups, and “survminer” package (version 0.4.9) [33] of R project was used for showing survival analysis results.

In order to study whether the overall expression of 7 m6A cross-talk genes had an impact on survival, the expression values of 7 genes were used for multivariate analysis followed by univariate analysis. Firstly, 7 variable genes were used to establish COX-PH for multivariate analysis, and then the risk scores of each gene in all tumor samples were obtained. According to the median risk scores of the samples, the samples were divided into a high-risk group and a low-risk group for survival analysis. According to the results (HR, 95% CI, *p* value) obtained by univariate and multivariate cox regression analysis, two forest plots were plotted by using the ggplot2 package (version 3.3.3) [34] in the R program (version 3.6.3). Every HR (hazard ratio) represents a relative risk of death that compares one instance of a binary feature to the other instance reference. An HR>1 indicates an increased risk of death, while an HR<1 represents a decreased risk of death.

A predictive nomogram for prostate cancer by combining the expression values of seven m6A methylation regulator genes with clinical variables has not been reported. Therefore, we constructed a prognostic nomogram by integrating clinical factors and gene expression using the TCGA-PRAD dataset. The nomogram was plotted by using the rms package (version 6.2-0) [35] and survival package (version 3.2-10) [32] in R program (version 3.6.3) and by following the Cox regression statistical analysis. Overall survival (OS) was chosen as the prognostic type. The included variables were T stage, N stage, M stage, primary therapy outcome, race, age, residual tumor, and PSA (ng/ml), as well as the expression of seven m6A methylation regulator genes. The number of M1 subgroup (*n*(M1) = 3) of the M stage variable is too less and therefore cannot be included to be the analysis of nomogram plot.

### 2.8. Functional Analysis and Gene Filter Analysis of m6A-Related Cross-Talk Gene

To analyze the biological functions regulated by m6A-related cross-talk gene, the clusterProfiler package (version 3.15) [36] of R project was used for conducting GO Biological process and KEGG pathway analysis. In order to further screen m6A-related cross-talk gene that plays a regulatory role in PRAD and PD, the LASSO regression model was built to screen these 7 m6A-related cross-talk genes. Firstly, the expression values of 7 m6A-related cross-talk genes in PRAD and PD samples were extracted, including the case and control groups. Based on these two groups, LASSO models were established to acquire the key m6A-related cross-talk gene with PRAD and PD dataset, respectively. After obtaining the key m6A cross-talk genes of PRAD and PD, respectively, the intersection genes, which are the risk m6A cross-talk genes that are more critical for regulating PRAD and PD, were obtained.

### 2.9. Protein-Protein Interaction Network for m6A-Related Cross-Talk Genes

The m6A-related cross-talk genes screened by LASSO analysis and those obtained by univariate analysis were combined, and then the expression values of the combined genes in disease samples in PRAD and PD were obtained. The high confidence level regulation m6A gene for PRAD in the RMVar database (https://rmvar.renlab.org/) [37] was extracted; afterward, the genes expression in PRAD was obtained. The correlation between the predicted m6A gene obtained by RMVar database and the cross-talk genes were analyzed; and the GENIE3 package (version 3.15) [38] of R project was used to calculate the weight relationship among genes. The predicted results according to the weight value were sorted, and the relationship pairs with the TOP 25% of the weight were screened as the high confidence level of the protein interaction pair. Then, the genes in these high confidence level relationship pairs and their expression values in PD were extracted, and the GENIE3 package (version 3.15) was used to predict the weight of these genes based on PD dataset. The TOP 25% relationship pair as the final Target _m6A_*-*Other_m6A_ PPI was acquired.

In addition, to analyze the role of m6A-related genes in the entire biological network, the protein-protein interaction (PPI) relationship pairs of m6A-related gene interactions were obtained from the Human Protein Reference Database (HPRD) (http://www.hprd.org/index_html) [39] and BIOGRID (http://thebiogrid.org/) [40]. Then, the expression values in PRAD and PD for the interacted gene were obtained, and the high confidence level pairs (Target _m6A_-Other_non-m6A_ PPI) with the GENIE3 package of R project were acquired. Finally, the Target _m6A_*-*Other_m6A_ PPI and Target _m6A_-Other_non-m6A_ PPI were combined to build a PPI network regulated by m6A-related cross-talk gene using Cytoscape software.

## 3. Results

### 3.1. Data Preprocessing

**Figure 2:**
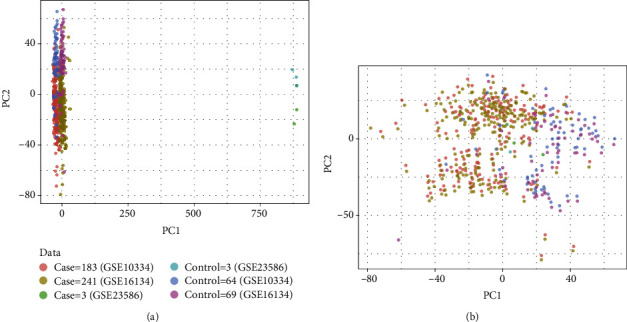
PCA analysis results of PD batches before (a) and after (b) rectification.

After merging all the samples in the three datasets related to PD according to their common genes, then the ComBat method in the sva package of R was used to perform batch correction on the combined data and perform PCA analysis on the corrected results (Figure 2). The results show that there is a certain difference between the three datasets without correction, and this difference has been significantly reduced after correction.

### 3.2. Differential Expression Analysis

**Figure 3:**
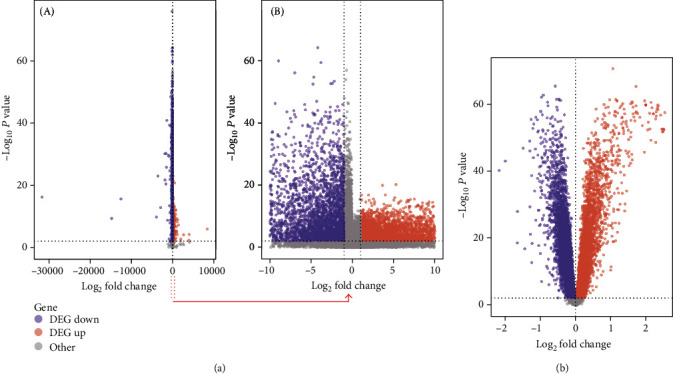
Volcano plot of DEGs in PRAD (a) and PD (b). (a-A) shows all DEGs deregulated in PRAD, (a-B) shows the |*logFC*| ≤10 of DEGs deregulated in PRAD. (b) shows all DEGs deregulated in PD.

For the PRAD, the genes with *p* value <0.01, |log2(FC)| ≥1 were regarded as differentially expressed genes (DEG), where genes with log2(FC) ≥1 indicates upregulated genes, and genes with log2(FC) ≤-1 indicates downregulated genes. For the PD dataset, the genes with *p* value<0.01 and |log2(FC)| >0 are acted as differentially expressed genes, where log2(FC) >0 was an upregulated gene and log2(FC) <0 was a downregulated gene. The counts of DEG are shown in Table 3. 8,855 DEGs including 4,500 upregulated DEGs and 4,355 downregulated DEGs were identified to be deregulated in prostate cancer. 14,348 DEGs consisting of 7,153 upregulated DEGs and 7,195 downregulated DEGs were deregulated in periodontitis.  Figure 3 uses volcano plots to show the differential expression of DEGs in prostate cancer (Figure 3(a)) and periodontitis (Figure 3(b)).

### 3.3. m6A-Related Cross-Talk Gene between in PRAD and PD

**Figure 4:**
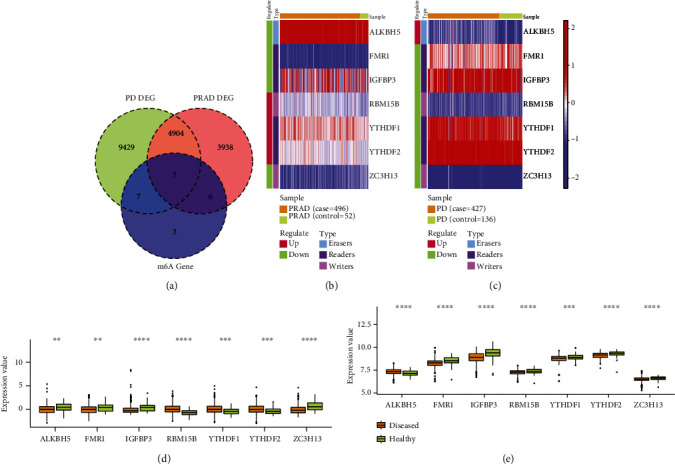
The gene expression of m6A-related cross-talk gene. (a) The relationship among DEG of PRAD, DEG of PD, and m6A genes; (b and c) the gene expression of m6A-related cross-talk genes in PRAD and PD; (d and e) the expression difference of m6A-related cross-talk genes between disease and normal sample in PRAD and PD. The corresponding relationship between the *p* value and the “∗” sign is ns: *p* > 0.05, ^∗^*p* ≤ 0.05, ^∗∗^*p* ≤ 0.01, ^∗∗∗^*p* ≤ 0.001, and ^∗∗∗∗^*p* ≤ 0.0001.

The common genes among 4855 DEGs of PRAD, 14348 DEGs of PD, and 23 m6A-related genes were obtained (Figure 4(a)). 7 common genes (ALKBH5, FMR1, IGFBP3, RBM15B, YTHDF1, YTHDF2, and ZC3H13) were acquired which was acted as the m6A-related cross-talk gene. We extracted the expression values of 7 m6A-related cross-talk genes in PRAD and PD datasets and demonstrated the expression of these genes in PRAD and PD by using pheatmap package of R project (Figures 4(b) and 4(c)).

In addition, the Wilcoxon test was used to examine the differences of 7 m6A-related cross-talk genes between the disease group and the normal group (Figures 4(d) and 4(e)). The smaller the *p* value of the test result, the more significant the sample comparison result. Figures 4(d) and 4(e) showed that the 7 m6A-related cross-talk genes were differentially expressed in both the diseased and healthy control groups.

Table 4 shows the expression pattern of 7 m6A-related cross-talk genes in diseased samples compared with healthy control samples. This table also shows whether the expression patterns of 7 crosstalk genes were consistent between periodontitis and prostate cancer. Table 4 shows that the expression patterns of three genes (FMR1, IGFBP3, and ZC3H13) in prostate cancer and periodontitis were consistent by showing their downregulation in diseased samples compared with healthy control samples. However, the expression patterns of the other four genes (ALKBH5, RBM15B, YTHDF1, and YTHDF2) in prostate cancer and periodontitis were not consistent.

### 3.4. Tumor Mutation Burden (TMB) Analysis of m6A-Related Cross-Talk Genes in PRAD

**Figure 5:**
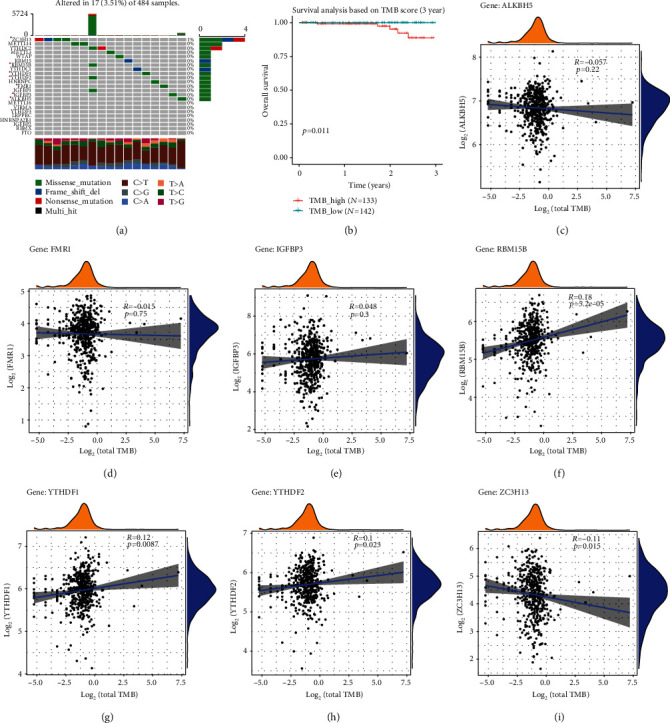
Tumor mutation burden (TMB) analysis related of m6A-related cross-talk genes in PRAD. (a) The mutation for m6A-related genes in PRAD. The horizontal axis represents samples, and the vertical axis represents genes; (b) survival status of TMB high and low group; (c–i) correlation analysis between TMB with ALKBH5, FMR1, IGFBP3, RBM15B, YTHDF1, YTHDF2, and ZC3H13.

After obtaining PRAD's Simple Nucleotide Variation dataset from TCGA, the mutation of 23 m6A regulator genes with maftools of R project was observed (Figure 5(a)), and 7 m6A-related cross-talk genes were found. It can be seen that the 7 m6A-related cross-talk genes are mutated. Then the Simple Nucleotide Variation dataset is calculated to obtain TMB score. All samples are divided into a high score group and a low score group according to the median of TMB score. Survival analysis was performed on the grouped samples (Figure 5(b)). As can be seen from Figure 4(b), the survival rate of the high score group was lower than that of the low score group with the increase of time, indicating that tumors may have a greater impact on the whole body in patients with high TMB scores. Meanwhile, the expression values of 7 m6A-related cross-talk genes in PRAD samples were obtained, and Pearson correlation analysis was conducted on these genes combined with TMB scores of the samples (Figures 5(c)–5(i)). The results showed that RBM15B, YTHDF1, and YTHDF2 were highly positively correlated with TMB, while ZC3H13 was negatively correlated with TMB.

### 3.5. Survival Analysis of m6A Cross-Talk Gene

**Figure 6:**
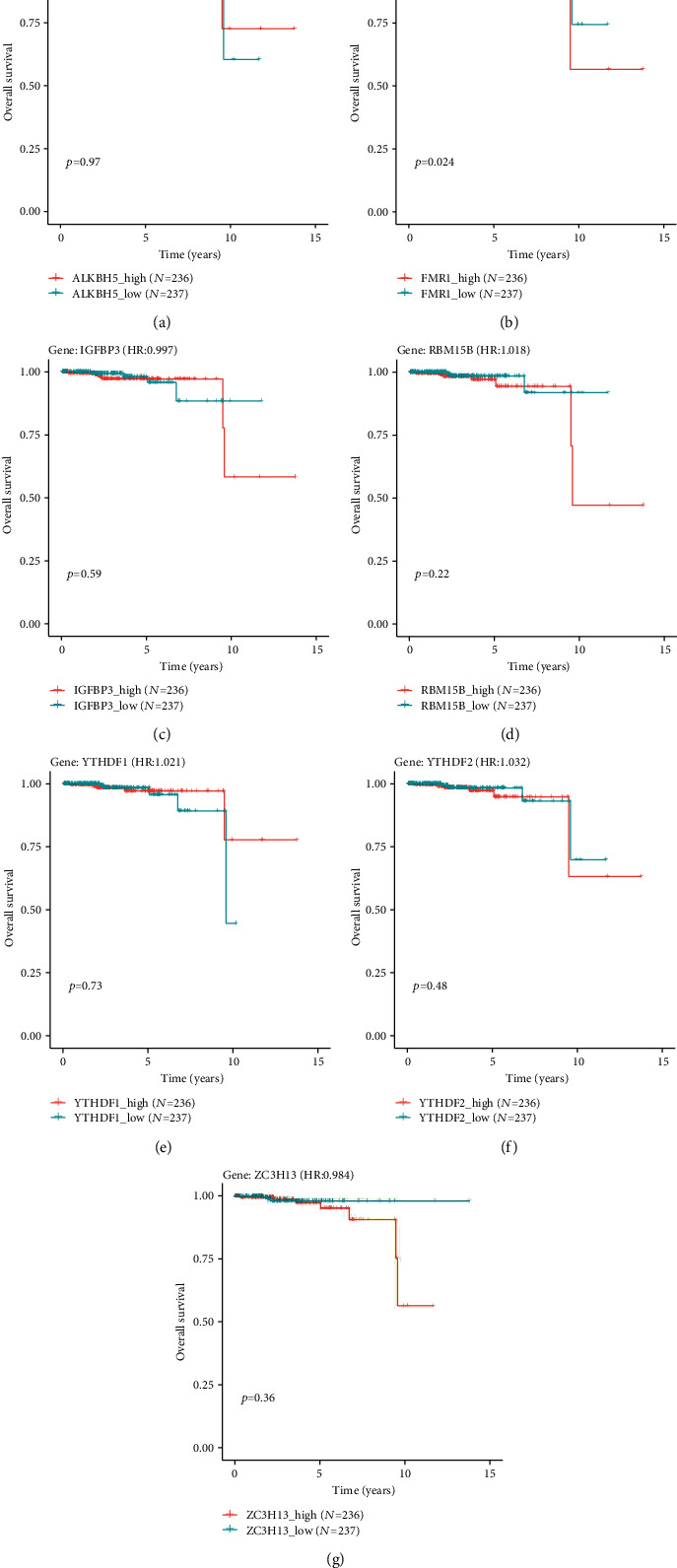
Univariate survival analysis of seven m6A-related cross-talk genes.

The expression values of 7 m6A-related cross-talk genes were obtained from PRAD tumor samples and then established COX-PH model for univariate analysis of each gene. Risk scores were obtained for all samples based on univariate analysis, and the samples were divided into a high-risk and low-risk groups by median risk scores, followed by survival analysis (Figures 6(a)–6(g)). As can be seen from Figure 6, FMR1 was significant in survival and had a good prognosis, while other genes had no significant effect on survival.

**Figure 7:**
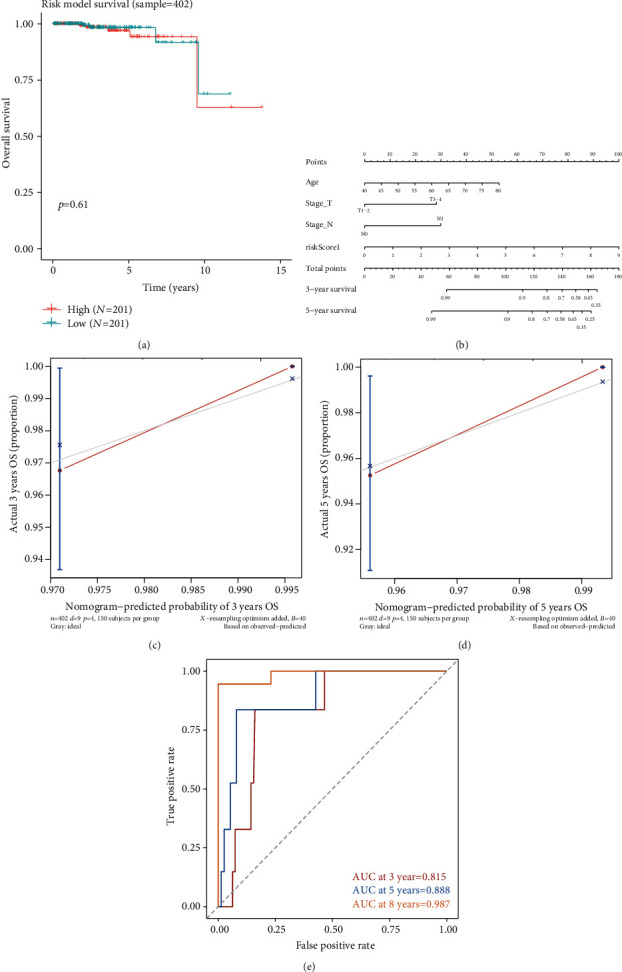
(a) Kaplan-Meier curves of seven m6A-related cross-talk genes in PRAD using multivariate COX-PH model; (b) nomogram of pathological features and survival relationship; (c–d) a calibration curve of nomogram for 3 years and 5 years; (e) ROC curve with time dependence.

To investigate whether the overall expression of 7 m6A-related cross-talk genes influenced survival, the multivariate analysis of these genes was performed by using a COX-PH model. According to the median of risk score, the samples were divided into a high-risk group and a low-risk group for survival analysis (Figure 7(a)). As can be seen from Figure 7(a), the survival analysis effect of multivariate Cox model is not significant. In addition, tumor samples of PRAD were grouped according to their pathological characteristics, and then the survival analysis with the 7 m6A-related cross-talk genes was performed. The sample risk score and pathological features obtained from multivariate analysis were combined to examine the sample risk and the effect of pathological features on survival. A COX-PH model was built using “rms” package of R project, and nomograms were plotted to see the relationship between pathological features and survival (Figure 7(b)). From Figure 7(b), it can be obtained that age, Stage_T, and Stage_N have an impact on survival, and the influence of age on survival is larger.

Clinical information and corresponding sample numbers are shown in Table 5. In Table 5, sex and Stage_M pathological features are not used because the difference between the groups with the two feature was too great. A total of 402 valid samples were obtained from the five pathological features of OS, OS_Event, age, Stage_T, and Stage_N.

In order to verify the prediction effect of COX-PH model established by RMS package, three methods were used to verify. Firstly, the calibrate method in the rms package was used to calculate and draw a diagram to show the calibration curve of the model for 3 years and 5 years (Figures 7(c) and 7(d)). The result showed that the similar between the model predicted results and the sample true results are more than 95%, and the nomogram had favorable predictive power for the 3-year and 5-year survival of patients with PRAD. The C-index value of the model was also calculated, and the result shows that C-index value is 0.8064 and the model has a good effect. Finally, the timeROC package of R project is used to calculate the ROC of the model for 3, 5, and 8 years. Based on the ROC curve (Figure 7(e)), it can be seen that the model prediction accuracy is good in the 3-period survival time range.

**Figure 8:**
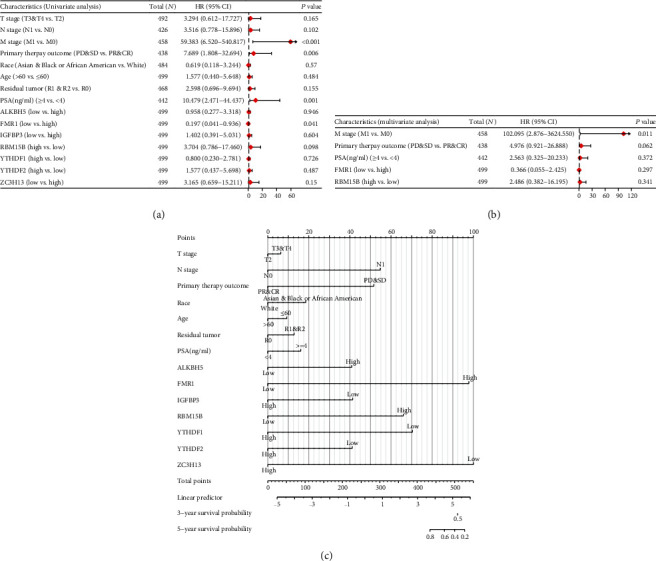
The forest plot and nomogram plot showing the relationship between seven m6A methylation genes and overall survival of prostate cancer. (a) The forest plots showing the univariate regression analyses results of seven m6A methylation regulator genes and clinicopathologic parameters with overall survival (OS) in prostate cancer patients. (b) The forest plots showing the univariate regression analyses results of seven m6A methylation regulator genes and clinicopathologic parameters with overall survival (OS) in prostate cancer patients. (c) The nomogram plot for predicting probability of patients with 1-, 3-, and 5-year overall survival based on the various clinicopathological features and the expression levels of seven m6A methylation genes.

Table 6 and Figure 8(a) shows that a hazard ratio of 0.197 for FMR1 low expression group means that prostate cancer patients who were detected with the low expression of FMR1 gene have a decreased risk of death compared to prostate cancer patients who were detected with the high expression of FMR1 gene (*p* = 0.041). The results of univariate Cox regression analysis indicated that several factors (e.g., M0 stage (*p* < 0.001), primary therapy outcome (PR&CR) (*p* = 0.006), PSA ≥4 ng/ml (*p* = 0.001), and FMR1 high expression (*p* = 0.041 < 0.05)) were negative predictor for overall survival outcome in prostate cancer patients; however, the other six genetic factors were not shown to be significant predictors for the overall survival outcome in prostate cancer patients.

Table 7 and Figure 8(b) show the results of the multivariate Cox regression analysis, indicating that M1 stage (*p* = 0.011) was negative predictor for overall survival outcome in prostate cancer patients; however, the seven genetic factors were not shown to be significant predictors for the overall survival outcome in prostate cancer patients.

A nomogram plot (Figure 8(c)) was constructed to predict the 1-, 3-, and 5- year survival probability of prostate cancer patients by integrating the expression level of and independent clinical variables. Total points were calculated by adding the points of the genetic score, age, and TNM stage. A worse prognosis was represented by a higher total number of points on the nomogram.

**Figure 9:**
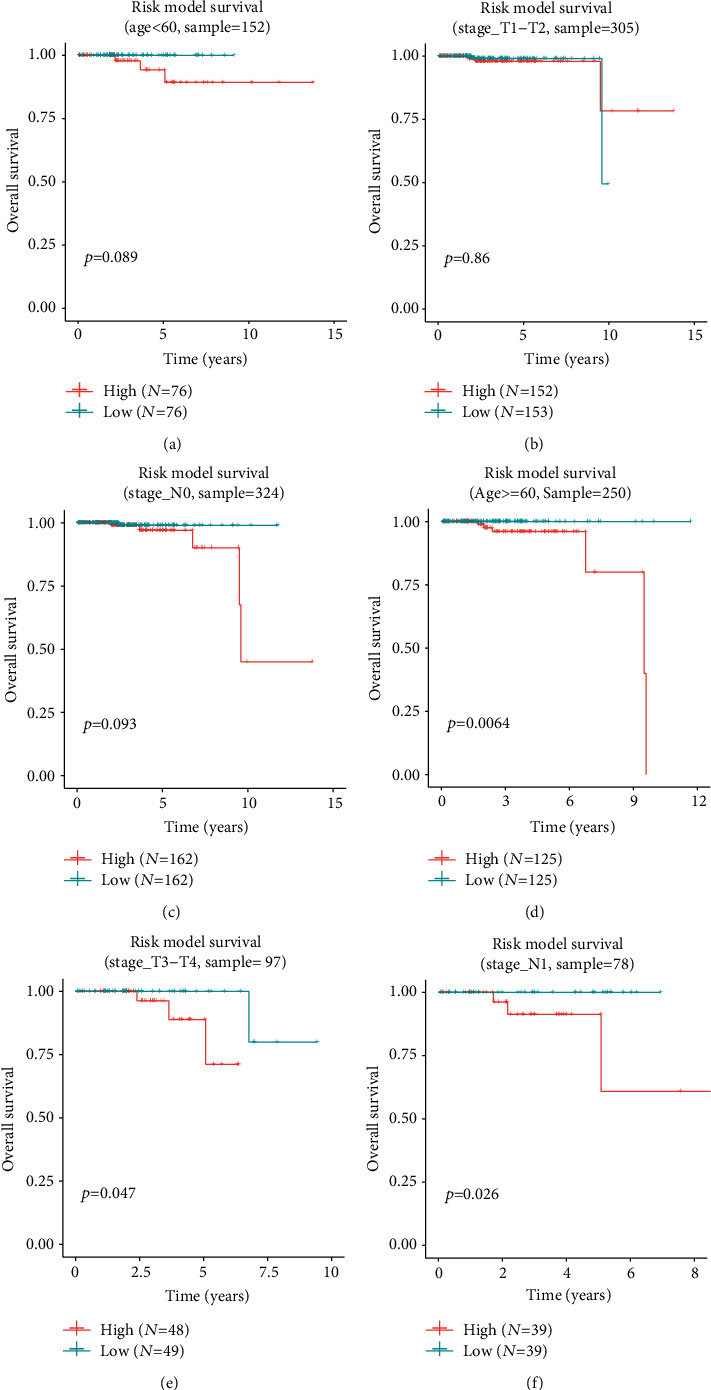
Survival curves grouped for different clinicopathological features. (a) age< 60; (b) stage T1-T2; (c) stage N0; (d) age ≥ 60; (e) stage T3-T4; (f) stage N1.

Through model evaluation, it was found that age, Stage_T, and Stage_N pathological characteristics all had certain influence on survival. Therefore, we first grouped the samples under the 7 m6A-related cross-talk genes according to different pathological characteristics, and the grouped samples were analyzed by using the survival package of R project for COX-PH model. The samples were divided into high-risk groups and low-risk groups for survival analysis according to the median risk score of the samples (Figures 9(a)–9(f)). The results showed that the survival of 7 m6A-related cross-talk gene was significant in age ≥60, STAGE_T3-T4, and Stage_N1 groups, and the survival rate of the high-risk group was lower than that of the low-risk group.

### 3.6. Functional Analysis and Gene Filter Analysis of m6A-Related Cross-Talk Gene

**Figure 10:**
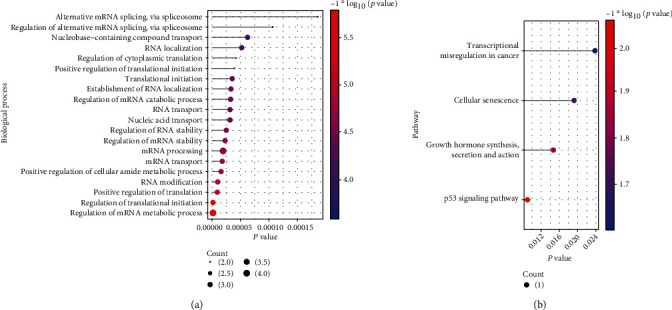
The m6A-related cross-talk gene was significantly enriched in (a) biological process and (b) pathway.

With the clusterProfiler of R project, the m6A-related cross-talk genes were found to be enriched into several GO biological process and KEGG pathways, and the functions with *p* value<0.05 were significant (Figures 10(a)–10(b)). The results showed that m6A-related cross-talk gene mainly regulated the regulation of mRNA metabolic process, regulation of translational initiation, and RNA modification (Figure 10(a)). Meanwhile, m6A-related cross-talk gene takes part in p53 signaling pathway, growth hormone synthesis, secretion and action, cellular senescence, and transcriptional misregulation in cancer (Figure 10(b)).

**Figure 11:**
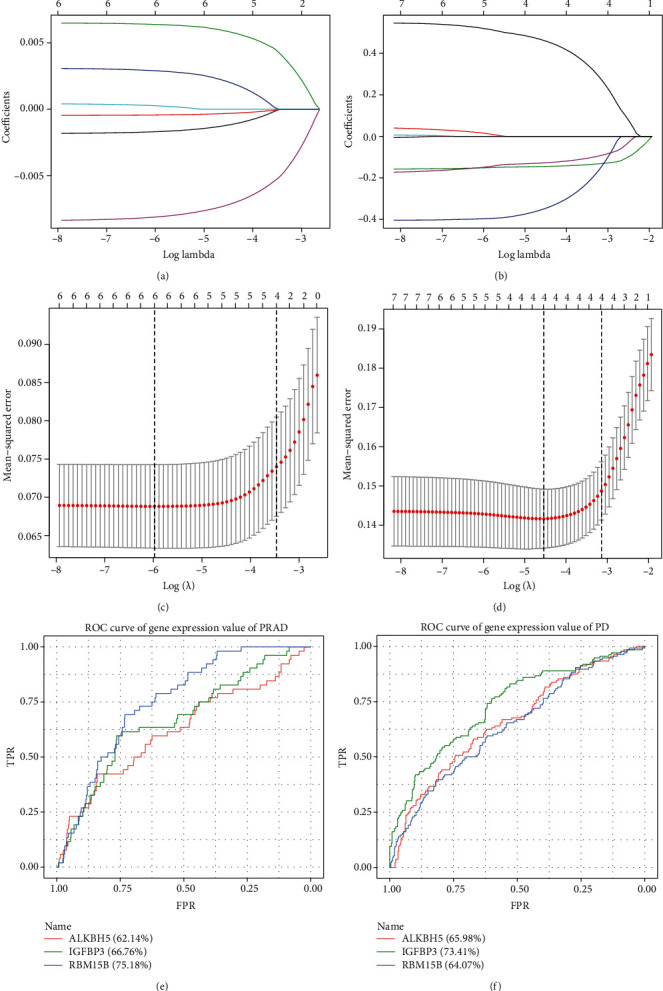
The risk m6A-related cross-talk genes in PRAD and PD. (a) and (b) are the results of LASSO analysis. Each line in the figure represents gene. As the gene approaches 0, the larger the *x*-coordinate (Log Lambda) is, the more critical the gene is. (c) and (d) are the results of model cross-validation. There are two dashed lines in the figure, one is lambda.min for the minimum mean square error and the other is lambda.1se for the standard error from the minimum mean square error. (e) and (f) are ROC results of 3 genes in PRAD and PD.

To further screen m6A-related cross-talk genes that play a key role in both PRAD and PD, lasso regression analysis was used for screening. First, the expression values of 7 m6A-related cross-talk genes were extracted in PRAD and PD. Then based on the disease and normal group, the LASSO model was established to analyze PRAD and PD, respectively (Figures 11(a)–11(d)). Finally, four genes (ALKBH5, IGFBP3, RBM15B, and ZC3H13) were obtained in PRAD and four genes (ALKBH5, IGFBP3, RBM15B, and YTHDF2) were obtained in PD. Three genes were both screened in PRAD and PD, which were ALKBH5, IGFBP3, and RBM15B. The expression values of these 3 genes in PRAD and PD were obtained, and ROC analysis was performed to analyze the prediction accuracy of the genes (Figures 11(e) and 11(f).

### 3.7. Protein-Protein Interaction Network for m6A-Related Cross-Talk Genes

From the RMVar database, 409 predicted PRAD-related m6A genes were obtained. After lasso analysis and univariate survival analysis, we finally obtained four risk m6A-related cross-talk genes (ALKBH5, IGFBP3, RBM15B, and FMR1). To predict the correlation between four key m6A genes and the predicted m6A gene of PRAD, the expression values of these genes from PRAD were extracted, and then the weight relationship between them was predicted by using GENIE3 packages of R project. The weights in descending order were sorted, and then the TOP 25% of the relationship pairs (PPI_pair1) and the genes in the relationship pairs (gene_list1) were obtained. The expression values of the gene_list1 in PD were obtained, and then the GENIE3 package was used to predict the weights of the 4 m6A genes and gene_list1. The relationship pairs in PD in descending order were obtained, and the relationship pairs with a weight of TOP 25% (PPI_pair2) were filtered. The PPI_pair1 and PPI_pair2 common relationship pairs were obtained, and then 152 relationship pairs (Target _m6A_*-*Other_m6A_ PPI) were acquired.

**Figure 12:**
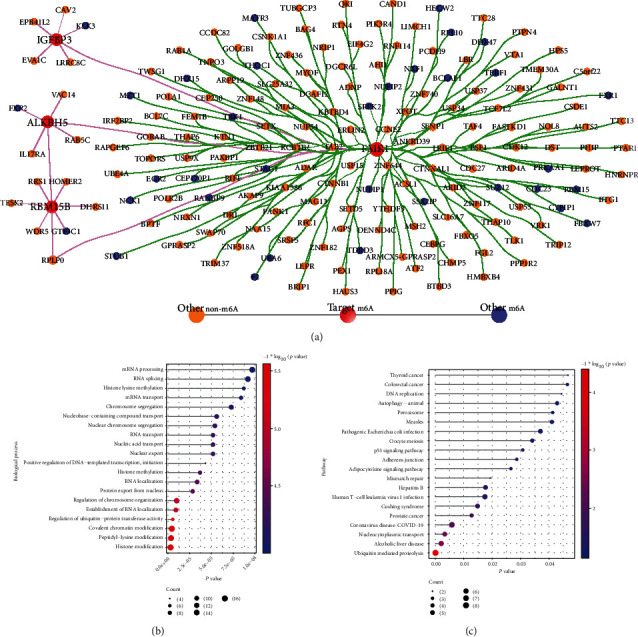
The PPI networks and functional enrichment regulated by m6A-related cross-talk genes. (a) The PPI network for m6A-related cross-talk genes; (b) the biological process enrichment for whole PPI network; (c) the significant pathway for whole PPI network of m6A-related cross-talk genes enriched.

In addition, the correlations from HPRD and BIOGRID and combined gene expression correlation in PRAD and PD were derived, and 39 PPI relationship pairs (Target _m6A_-Other_non-m6A_ PPI) for m6A-related gene interactions were obtained. The Target _m6A_*-*Other_m6A_ PPI and Target _m6A_-Other_non-m6A_ PPI were combined to construct the PPI network of m6A-related cross-talk genes, which included 183 nodes and 185 edges (Figure 12(a)).

From the network, FMR1 interacts with more proteins throughout the network and is highly correlated with the other 3 m6A-related cross-talk genes (ALKBH5, IGFBP3, and FMR1). FMR1 affects more biological functions by regulating interacting proteins.

From the m6A cross-talk genes-related PPI network, a total of 183 genes were obtained. These 183 genes interact with each other and play a potential regulatory role in the biological functions associated with PRAD and PD. To this end, the clusterProfiler package of R project was used to perform the function enrichment of 183 genes and *p* value <0.05 as a significant function. The results showed that 183 genes significantly regulated histone modification, peptidyl-lysine modification, regulation of ubiquitin-protein transferase activity, and so on (Figure 12(b)). Moreover, 183 genes involved in the ubiquitin-mediated proteolysis, mismatch repair, p53 signaling pathway, and DNA replication (Figure 12(c)).

## 4. Discussion

Seven m6A-related cross-talk genes (*ALKBH5*, *FMR1*, *IGFBP3*, *RBM15B*, *YTHDF1*, *YTHDF2*, and *ZC3H13*) were differentially expressed in both PC and PD. These genes were significantly enriched in several signaling pathways, such as the pathways involved in pathogenic *Escherichia coli* infection, nucleocytoplasmic transport, ubiquitin-mediated proteolysis, *p53* signaling, growth hormone synthesis, secretion, and action, cellular senescence, and transcriptional misregulation in cancer. Except for *RBM15B*, the other six cross-talk genes examined in the current study have been strongly studied in previous literature.

AlkB homolog 5 (*ALKBH5*), also known as m6A demethylase, provides the eraser function for m6A methylation by mediating m6A methylation reversal [41, 42]. *ALKBH5* expression was significantly lower in healthy controls compared to patients with PC. Patients with castration-resistant prostate cancer (CRPC) with bone metastasis also showed less *ALKBH5* downregulation compared to patients with CRPC with lymph node metastasis. Additionally, *ALKBH5* was found to be negatively related to the Gleason score [43], which supports *ALKBH5*'s role as a prognostic indicator for PC [43].

According to the current study, *ALKBH5* mRNA expression was not related to overall survival in PC (Figure 5(a)). However, a previous bioinformatics study found that patients with PC with a copy number gain of *ALKBH5* had worse relapse-free survival (RFS) rates. This indicates that *ALKBH5* copy number variation patterns is significantly associated with RFS rates in PC [44]. In comparison, *ALKBH5* was found to be upregulated in PD samples compared to healthy controls. A recent study found that monocytic infiltration in PD was directly proportional to *ALKBH5* expression, indicating a positive correlation between *ALKBH5* and monocyte levels in PD [45].

The expression of fragile X mental retardation (*FMR1*) protein was downregulated in both PD and PC (Table 3). Adamsheck et al. found that men with low CGG repeat lengths in the *FMR1* gene had significantly higher rates of PC in the family (*p* = 0.007) [46]. However, there is minimal research on the role of *FMR1* in regulating cancer biology. Another study demonstrated that exosomal *FMR1-AS1* played a role in maintaining the dynamic interconversion state of cancer stem-like cells in female esophageal carcinoma through the activation of the TLR7-NF*κ*B signaling axis. However, there is still no research regarding the role of the *FMR1* gene in regulating PC [47].

The current study showed that there was a significant association between *FMR1* and overall survival in PC (*p* = 0.024, Figure 5(b)). The prognostic value of *FMR1* was also determined for other cancers, such as esophageal squamous cell carcinoma [48], glioma [49], and aggressive breast cancer [50]. While there is still no information on the role of the *FMR1* gene in PD, Muzzi found that FMR1-associated fragile X syndrome is not significantly associated with PD [51, 52]. A previous study demonstrated that overexpression of *FMR1* protected cardiomyocytes against lipopolysaccharide-induced myocardial injury by reducing oxidative stress and apoptosis [53]. This supports the theory that *FMR1* upregulation may be a novel strategy for PD treatment.

Research has also implicated the insulin-like growth factor pathway in the development of PC. Insulin-like growth factor-binding protein-3 (*IGFBP*-3) is a potent apoptotic molecule that is regulated by p53. It is significantly suppressed in various types of cancers, including PC [54]. Previously, Perry *et al.* reported that *IGFBP-3* methylation may play a role in early PC development [55]. A large prospective cancer screening trial found that there was no association between *IGFBP-3* and the risk for PC [56]. Seligson *et al.* conducted a study on 226 patients and found that high nuclea*r IGFBP-3* expression was a very strong predictor of cancer recurrence in patients with low-grade PC [57]. Another study supported this finding by demonstrating that prediagnostic *IGFBP-3* levels (Hazard Ratio = 0.93; 95%Confidence Interval = 0.65–1.34) were not associated with overall survival outcomes in PC. The current research similarly supports these findings (*p* = 0.59, Figure 5(c)). The current study also used GEO datasets to analyze the role of *IGFBP-3* in PD. The upregulation of *IGFBP-3* in human gingival fibroblasts increased IGF transport and enhanced periodontal wound healing and regeneration [58]. There was no correlation between *IGFBP-3* levels in gingival crevicular fluid with periodontal parameters (e.g., probing depth and gingival index) [59].

The *YTHDF* family genes (*YTHDF1*, *YTHDF2*, and *YTHDF3*) can recognize bases undergoing m6A methylation, as well as bases participating in downstream translation, mRNA degradation, and mRNA exit rate acceleration [60]. Previous studies have demonstrated that PC samples expressed higher levels of *YTHDF1* and *YTHDF2* mRNA compared to healthy tissue [61, 62]. Upregulation of *YTHDF1* and *YTHDF2* promotes PC cell proliferation, invasion, and migration [61, 62]. In particular, *YTHDF2* modulated PC phenotypes by regulating the expression of its significantly positively correlated gene, tripartite motif-containing 44 (*TRIM44*) [61]. *YTHDF2* may exert this action by binding to m6A modification sites on tumor suppressor genes, such as *LHPP* and *NKX3-1*. This negatively affects mRNA levels, which promotes *AKT* phosphorylation-induced tumor progression [63]. To date, there is no data on the role of *YTHDF1* and *YTHDF2* in PD. However, *YTHDF2* knockdown in RAW264.7 cells, and primary bone marrow-derived macrophages does upregulate osteoclast-related gene expression and proinflammatory cytokine secretion [64]. It can be speculated that downregulation of *YTHDF2* enhances osteoclastic activity in PD, as well as promotes inflammation.

*ZC3H13* positively regulates latent membrane protein 1 (LMP1)-induced nuclear factor in kappa beta (NF-*κ*B) pathway activation [65, 66]. The potential role of *ZC3H13* in PD and PC is based on the role of the NF-*κ*B pathway in inflammation and cancer. The NF-*κ*B signaling pathway is one of the most strongly activated pathways in PD-derived inflammation [67, 68]. The activation of this pathway increases osteoclast mediated-periodontal bone resorption in PD [69]. The constitutive activation of NF-*κ*B transcription factors promotes tumor-cell survival by inhibiting the apoptosis of PC cells [70]. NF-*κ*B nuclear expression also strongly predicted biochemical recurrence following radical prostatectomy with positive surgical margins. As such, NF-*κ*B nuclear expression can be regarded as an independent molecular marker for stratifying risk in patients with PC [71]. In addition, *ZC3H13* has been identified as an oncogene in kidney clear cell carcinoma. *ZC3H13* activates the NF-*κ*B signaling pathway in patients with this condition to promote tumor proliferation and invasion [72]. However, a separate study showed contrary evidence and identified *ZC3H13* as an upstream regulator of the Ras-ERK signaling pathway. In this study, *ZC3H13* suppressed colorectal cancer invasion and proliferation by inactivating Ras-ERK signaling [73]. There remains no data on the role of *ZC3H13* in PC pathogenesis.

Among the signaling pathways activated by the seven cross-talk genes, the pathogenic *E. coli* infection pathway obtained our particular interest. *E. coli* in PD can produce genotoxic toxins that promote carcinogenesis. This potentially explains the epidemiologic data suggesting an increased risk for PC among patients with PD [74]. *E. coli* infection can increase the risk for PC through two proposed mechanisms. One mechanism involves bacterial migration from periodontal pockets to the peripheral blood and prostate tissue. *E. coli* was found in significantly large numbers in the expressed prostatic secretions and seminar fluid of subjects with PC compared to subjects with benign prostatic hyperplasia [75]. The other mechanism involves increased systemic inflammation secondary to *E. coli i*nfection in the peripheral blood of patients with PD. *E. coli* lipopolysaccharide (LPS)-stimulated peripheral blood mononuclear cells (PBMCs) obtained from the peripheral blood of subjects with chronic PD demonstrated higher levels of proinflammatory cytokine (TNF-*α* and IL-6) release compared with healthy subjects (*p* < 0.05) [76]. Increased levels of inflammatory markers in the peripheral bloodstream may promote carcinogenesis and a TME [77].

In summary, the majority of cross-talk genes identified in the current study played significant roles in the pathogenesis of PC and PD. This research has several limitations. First, the data analyzed in this study was derived from the gingival and cancer tissues of patients with PD and PC, respectively. The ideal analysis should utilize peripheral blood or PBMC samples; however, we were unable to obtain the GEO datasets of the peripheral blood samples of patients with PD, with and without PC. If we had such data, the genetic or methylation biomarkers obtained through sequencing assays may be used to evaluate the risk for PC in male patients with PD by examining the expression levels of these biomarkers in peripheral blood. Second, we were unable to validate the data in the current study. Additional experiments should be designed to examine the mRNA and methylation levels of the identified cross-talk genes in PD pathogen/*P. gingivalis*-derived LPS-stimulated PC cells compared with unstimulated PC cells. While this study has certain limitations, it still provided theoretical foundations for the potential genetic mechanisms that link PC and PD.

The current study can contribute to future research in several ways. From the viewpoint of precision medicine and drug development or repurposing, experimental and clinical studies that focus on these candidates may help identify shared susceptibility, exaggerated pathogenic mechanisms, genetic biomarkers, and potential therapeutic targets.

## 5. Conclusion

To conclude, seven m6A methylation regulator genes (*ALKBH5*, *FMR1*, *IGFBP3*, *RBM15B*, *YTHDF1*, *YTHDF2*, and *ZC3H13*) were identified as cross-talk genes that mediate the pathogenesis of PC and PD. These genes may be used to quantify the risk for PC among patients with PD, as well as be utilized as therapeutic targets for either condition.

## Figures and Tables

**Table 1:** PD and PRAD-related sample information.

	PRAD	PD		
Datasets	TCGA_PRAD	GSE10334	GSE16134	GSE23586
Platform	—	GPL570	GPL570	GPL570
Experimental	High-throughput sequencing	Array	Array	Array
Variation data	Known	Unknown	Unknown	Unknown
Clinical data	Known	Unknown	Unknown	Unknown
Case number	496	183	241	3
Control number	52	64	69	3
Total (sample)	548	247	310	6

**Table 2:** The detailed function of 23 m6A RNA methylation-related genes in RNA metabolism.

Name of 23 m6A methylation genes	Function in RNA metabolism
METTL3	Writers
METTL14	Writers
METTL16	Writers
WTAP	Writers
VIRMA	Writers
ZC3H13	Writers
RBM15	Writers
RBM15B	Writers
YTHDC1	Readers
YTHDC2	Readers
YTHDF1	Readers
YTHDF2	Readers
YTHDF3	Readers
HNRNPC	Readers
FMR1	Readers
LRPPRC	Readers
HNRNPA2B1	Readers
IGF2BP1	Readers
IGF2BP2	Readers
IGF2BP3	Readers
RBMX	Readers
FTO	Erasers
ALKBH5	Erasers

**Table 3:** DEG counts for PRAD and PD.

	PRAD	PD
DEG up	4500	7153
DEG down	4355	7195
Total DEG	8855	14348

**Table 4:** The expression pattern of 7 m6A-related cross-talk genes in diseased samples compared with healthy control samples.

	ALKBH5	FMR1	IGFBP3	RBM15B	YTHDF1	YTHDF2	ZC3H13
Prostate cancer	Downregulated	Downregulated	Downregulated	Upregulated	Upregulated	Upregulated	Downregulated
Periodontitis	Upregulated	Downregulated	Downregulated	Downregulated	Downregulated	Downregulated	Downregulated
If the expression patterns were consistent	Not consistent	Consistent, both downregulated	Consistent, both downregulated	Not consistent	Not consistent	Not consistent	Consistent, both downregulated

**Table 5:** Clinical information and corresponding sample numbers.

	Group	Sample(known)
Age	≥60	296
	<60	204

Sex	Male	500
	Female	0

OS	Within 3 years	292
	Within 5 years	413
	Overall survival	500

OS_Event	Alive	490
	Dead	10

Stage_T	T1-T2	382
	T3-T4	115

Stage_N	N0	348
	N1	79

Stage_M	M0	457
	M1	3

**Table 6:** The univariate survival analysis results regarding the seven m6A methylation regulator genes in predicting the overall survival risk of prostate cancer.

Characteristics (univariate analysis)	Total(N)	HR (95% CI)	*p* value
T stage (T3&T4 vs. T2)	492	3.294 (0.612-17.727)	0.165
N stage (N1 vs. N0)	426	3.516 (0.778-15.896)	0.102
M stage (M1 vs. M0)	458	59.383 (6.520-540.817)	<0.001
Primary therapy outcome (PD&SD vs. PR&CR)	438	7.689 (1.808-32.694)	0.006
Race (Asian & Black or African American vs. White)	484	0.619 (0.118-3.244)	0.570
Age (>60 vs. ≤60)	499	1.577 (0.440-5.648)	0.484
Residual tumor (R1&R2 vs. R0)	468	2.598 (0.696-9.694)	0.155
PSA(ng/ml) (≥ 4 vs. <4)	442	10.479 (2.471-44.437)	0.001
ALKBH5 (low vs. high)	499	0.958 (0.277-3.318)	0.946
FMR1 (low vs. high)	499	0.197 (0.041-0.936)	0.041
IGFBP3 (low vs. high)	499	1.402 (0.391-5.031)	0.604
RBM15B (high vs. low)	499	3.704 (0.786-17.460)	0.098
YTHDF1 (high vs. low)	499	0.800 (0.230-2.781)	0.726
YTHDF2 (high vs. low)	499	1.577 (0.437-5.698)	0.487
ZC3H13 (low vs. high)	499	3.165 (0.659-15.211)	0.150

**Table 7:** The multivariate survival analysis results regarding the seven m6A methylation regulator genes in predicting the overall survival risk of prostate cancer.

Characteristics (multivariate analysis)	Total (*N*)	HR (95% CI)	*p* value
M stage (M1 vs. M0)	458	102.095 (2.876-3624.550)	0.011
Primary therapy outcome (PD&SD vs. PR&CR)	438	4.976 (0.921-26.888)	0.062
PSA (ng/ml) (≥ 4 vs. <4)	442	2.563 (0.325-20.233)	0.372
FMR1 (low vs. high)	499	0.366 (0.055-2.425)	0.297
RBM15B (high vs. low)	499	2.486 (0.382-16.195)	0.341

## Data Availability

The datasets used and/or analyzed during the current study are available from the corresponding author on reasonable request.
